# Vascular profiling of the spinal cord and cortex reveals region-specific endothelial dysfunction and fibroblast remodeling in human ALS

**DOI:** 10.1186/s40478-026-02449-x

**Published:** 2026-10-10

**Authors:** Kuo Liao, Jianing Lin, Yangfan Zhou, Sebastian A. Lewandowski

**Affiliations:** 1 Department of Clinical Neuroscience, Karolinska Institutet, Stockholm, Sweden; 2 Center for Molecular Medicine, Karolinska University Hospital, Stockholm, Sweden

**Keywords:** ALS, Blood–brain barrier, Vasculature, Endothelial cell, Fibroblast, snRNA-seq

## Abstract

Amyotrophic lateral sclerosis (ALS) is a fatal neurodegenerative disorder characterized by progressive degeneration of upper and lower motor neurons. Increasing evidence suggests that neurovascular dysfunction contributes to ALS pathogenesis, but a comprehensive understanding of vascular cell alterations in the human central nervous system remains lacking. Here, to systematically characterize neurovascular changes in ALS, we curated single-nucleus transcriptomic datasets from human spinal cord and cortical tissues with a specific focus on vascular cell populations. Comparative analyses revealed substantial vascular perturbations in the spinal cord compared to the cortex. Among vascular-associated cell populations, endothelial cells and fibroblasts exhibited the most pronounced transcriptional alterations. Endothelial cells from ALS spinal cords displayed reduced blood–brain barrier integrity signatures and increased expression of inflammatory and stress-response programs, with the greatest changes manifested in capillary endothelial cells. In parallel, we generated a refined cellular atlas of spinal cord fibroblasts and identified three major fibroblast populations, including a distinct *LAMA1*^low^ perivascular fibroblast subtype. Fibroblast signatures were found to be consistently elevated in ALS across single-nucleus transcriptomic, bulk transcriptomic, and experimental validation datasets. Together, our findings establish a region-resolved view of neurovascular alternations in human ALS, which is characterized by endothelial barrier dysfunction and disease-associated remodeling of specialized perivascular fibroblasts. These findings broaden the cellular framework of ALS pathogenesis beyond neuronal and glial alterations and highlight the neurovascular niche as an integral component of human ALS disease biology.

**Supplementary Information:**

The online version contains supplementary material available at https://doi.org/10.1186/s40478-026-02449-x.

## Introduction

Amyotrophic lateral sclerosis (ALS) is a devastating neurodegenerative disorder characterized by the progressive degeneration of both upper and lower motor neurons [21]. It represents one of the most common adult-onset motor neuron diseases worldwide, with an estimated lifetime risk of approximately 1 in 350 individuals [13]. Approximately 90% of ALS cases are classified as sporadic ALS (sALS), whereas fewer than 10% are familial ALS (fALS) [44]. Over the past decades, more than 30 ALS-associated risk genes have been identified, mainly including *TARDBP*, *C9orf72*, *SOD1*, and *FUS* [21]. This pronounced genetic and clinical heterogeneity poses a major challenge for the development of effective and broadly applicable therapies. Moreover, due to the lack of reliable early diagnostic biomarkers and disease-modifying treatments, ALS remains largely incurable. Most patients experience relentless functional decline and reduced quality of life, with a median survival of only 2–3 years after diagnosis [44].

The neurovasculature, which constitutes the structural basis of the blood–brain barrier (BBB) and the blood–spinal cord barrier (BSCB), plays a central role in maintaining central nervous system (CNS) homeostasis [29, 37]. The BBB/BSCB is a highly specialized and selective interface composed of endothelial cells, mural cells, perivascular cells, astrocytic end-feet, and the basement membrane, collectively forming the neurovascular unit [10]. This intricate system tightly regulates the exchange of molecules, ions, and immune cells between the circulation and the parenchyma [1]. Increasing evidence indicates that neurovascular dysfunction contributes to the pathogenesis of multiple neurodegenerative diseases [2]. In Alzheimer’s disease (AD), BBB disruption has been linked to impaired clearance of neurotoxic proteins such as amyloid-β, as well as exacerbated neuroinflammation [26]. Similarly, vascular abnormalities and increased BBB permeability have been reported in Parkinson’s disease (PD), potentially facilitating the infiltration of peripheral immune components and accelerating neuronal degeneration [3, 32]. Emerging studies suggested that vascular dysfunction also plays a role in ALS pathogenesis [56]. Structural alterations of the neurovascular unit, increased barrier permeability, and endothelial abnormalities have been observed in both ALS patients and animal models [16, 68]. In our previous work, we also underscored the altered perivascular fibroblast activities and perivascular space enlargement in ALS, which precede the disease onset [40].

The advent of single-cell or single-nucleus RNA sequencing (sc/snRNA-seq) has markedly advanced our ability to resolve cellular heterogeneity and construct high-resolution atlases of the CNS [48]. Vascular-focused applications of these technologies in other neurodegenerative diseases, particularly AD, have revealed extensive disease-associated transcriptional changes across multiple vascular cell types [73]. In ALS, several single-cell or single-nucleus transcriptomic datasets from human brain and spinal cord tissues have been generated in recent years, providing valuable resources for dissecting disease-associated cellular alterations [19, 35, 36, 42, 43, 49, 52, 62, 74]. However, these studies have primarily focused on neuronal, glial and immune populations, with relatively limited attention to vascular cell perturbations. Consequently, despite the availability of human ALS transcriptomic datasets, a curated vascular-focused analysis across CNS regions and vascular cell subtypes is still lacking. In particular, it remains unclear whether ALS-associated vascular perturbations are shared between the spinal cord and cortex or exhibit region-specific patterns, and which vascular cell populations are most prominently affected. Addressing these questions is essential for defining the cellular architecture of neurovascular pathology in human ALS.

In this study, we integrated multiple snRNA-seq datasets from the human spinal cord and cortex of ALS patients and controls to construct a curated vascular cell atlas. Based on this, we systematically characterized disease-associated changes across vascular cell types and CNS regions. This comparative framework revealed substantially more pronounced vascular perturbations in the spinal cord than in the cortex, with endothelial cells and fibroblasts showing prominent disease-associated alterations. We further identified capillary endothelial cells as a particularly affected population, characterized by reduced BBB/BSCB-associated signatures and transcriptional reprogramming toward a reactive state. In parallel, we resolved previously unrecognized heterogeneity among human spinal cord perivascular fibroblasts and identified a distinct *LAMA1*^low^ perivascular fibroblast subtype with a deeper perivascular localization and disease-associated remodeling in ALS. Together, our study establishes a region-resolved framework of the human ALS vasculature and highlights coordinated alterations of endothelial and fibroblast compartments as prominent features of neurovascular dysfunction in the ALS spinal cord.

## Materials and methods

### Vascular snRNA-seq atlas generation

Single-nucleus RNA sequencing (snRNA-seq) datasets from human ALS spinal cord and cerebral cortex were curated to construct a comprehensive vascular cell atlas. For the spinal cord dataset, raw count matrices were imported from 10× Genomics HDF5 files downloaded from Gene Expression Omnibus (GEO, accession number: GSE287257) [74]. Data were processed using Seurat (v5.3.0) [20]. Cells with fewer than 500 detected genes, more than 5000 detected genes, fewer than 1000 UMIs, or mitochondrial transcript proportions exceeding 15% were excluded. Individual samples were integrated using Harmony (v1.2.3) [31], followed by principal component analysis (PCA), graph-based clustering, and UMAP visualization. Vascular-associated clusters were identified based on the expression of canonical markers, including fibroblast markers (*COL1A1*, *COL1A2*, *BICC1*), endothelial markers (*CLDN5*), pericyte markers (*PDGFRB* and *RGS5*), and smooth muscle cell markers (*ACTA2*) [46, 52, 65].

To obtain a high-confidence vascular compartment, endothelial cells, fibroblasts, mural cells, and pericytes were extracted from the integrated spinal cord dataset. Low-quality outlier cells were removed using density-based spatial clustering of applications with noise (DBSCAN) on UMAP embeddings. UMAP coordinates were extracted from the Seurat object and subjected to DBSCAN clustering using the dbscan package (v1.2.2), with eps = 0.2 and minPts = 5. Cells assigned to cluster 0, corresponding to noise points not associated with any density-based cluster, were excluded as isolated outliers. Remaining vascular cells were normalized using SCTransform and integrated across donors using Harmony (v1.2.3). Clustering was performed on Harmony embeddings followed by UMAP dimensionality reduction. Cell identities were assigned according to established vascular markers and further refined into arterial, capillary, venous endothelial cells, pericytes, smooth muscle cells, and multiple fibroblast subpopulations. Mixed clusters exhibiting ambiguous marker expression (not expressing any major vascular cell type markers and no significant cluster-specific markers detected) were excluded from downstream analyses.

For the cortical vascular atlas, snRNA-seq datasets from ALS and non-neurological control donors were acquired from Synapse (Project SynID: syn51105515) and processed separately before integration [52]. Vascular cells annotated as “Vasc” in the original metadata were extracted and merged. Cells from donors represented by fewer than 100 vascular nuclei were excluded to minimize batch-correction artifacts. SCTransform normalization, PCA, and Harmony integration were performed using donor identity as the integration variable, followed by UMAP embedding and graph-based clustering. Contaminating immune and sample-specific clusters were removed through iterative re-clustering and marker-based inspection. Final cell-type annotations were assigned based on canonical vascular markers and classified into arterial, capillary, venous endothelial cells, pericytes, smooth muscle cells, perivascular fibroblasts, and meningeal fibroblasts.

### Vascular differential expression analysis across healthy and ALS groups

To characterize disease-associated transcriptional alterations within vascular cell populations, differential expression analyses were performed separately for spinal cord and cortical vascular atlases. For the spinal cord dataset, vascular cells were stratified according to disease status (ALS versus control). Differentially expressed genes (DEGs) were identified independently within each vascular cell type using the Seurat FindMarkers function with the Wilcoxon rank-sum test. Comparisons were performed between ALS and control nuclei with a minimum expression frequency of 10% (min.pct = 0.1) and a minimum log2 fold-change threshold of 0.05 (logfc.threshold = 0.05). Genes with adjusted *p*-values < 0.05 and absolute log2(fold change) > 0.5 were considered significantly differentially expressed. For the cortical dataset, differential expression analysis was conducted using an identical strategy. Within each vascular cell type, nuclei from ALS donors were compared with nuclei from pathologically normal (PN) controls using Seurat FindMarkers with the same filtering parameters (min.pct = 0.1, logfc.threshold = 0.05). Significant DEGs were defined as genes with adjusted *p*-values < 0.05 and absolute log2(fold change) > 0.5.

### BBB and periphery scoring in endothelial cells

To investigate endothelial functional alterations associated with ALS, endothelial cells were extracted from the spinal cord and cortical vascular atlases based on cell-type annotations. Endothelial populations were manually inspected on their UMAP projections to identify potential outliers. Cells that were originally annotated as endothelial cells but were spatially separated from the main endothelial clusters and instead localized within or adjacent to non-endothelial populations were considered potential contaminants and excluded from downstream analyses. To assess blood–brain barrier and peripheral endothelial associated characteristics, module scores were calculated using predefined gene signatures representing BBB-associated and peripheral endothelial programs [47].

**Table:** 

BBB gene module	*SPOCK2, SLCO1A2, SLC2A1, SLCO1C1, SLC22A8, ABCB1, ITM2A, LEF1, SLC7A5, ITIH5, BSG, MFSD2A, LRP8, CXCL12, PGLYRP1, ZIC3, SLC38A3, ABHD2, TFRC, APCDD1, SLC6A6, PROM1, APOD, CCDC141, SLC19A3, FLT1, PTN, FOXF2, SLC39A10, SPARCL1, ZIC2, SLC1A1, ATP10A, TNFRSF19, FOXQ1, FOSB, STRA6, SLC7A1, VWA1, PLTP, EFR3B, GPCPD1, SGPP2, HIAT1, IGF1R, OCLN, SLC16A4, ABLIM1*
Periphery gene module	*AQP1, IGFBP5, CD36, FABP4, GPIHBP1, MRC1, CYP4B1, MEIS2, EDNRA, SLC43A3, LYVE1, TSPAN7, BTNL9, MMRN1, SELP, HPGD, F8, PLVAP, LYZ, F2R, SERPINA3, PLPP3, NEURL3, CXCL9, IGFBP4, ESM1, PTGS1, CYTL1, HLA-DRA, CD300LG, GPR182, CCDC80, RELN, CD74, MLXIP, GIMAP4, ITGA9, LBP, PLPP1, LDB2, FSTL1, BACE2*

The signatures were adopted in their entirety from the original reference without modification and were intersected with the genes represented in our dataset before scoring. Gene module scores were calculated for each nucleus using Seurat’s AddModuleScore function, generating BBB and periphery module scores for downstream analyses.

Module scores were compared between ALS and control groups within endothelial subtypes and anatomical regions. Score distributions were visualized using violin and box plots, and statistical significance was evaluated using two-sided Wilcoxon rank-sum tests.

### Differential expression of capillary cells and gene ontology enrichment

Because capillary endothelial cells exhibited the largest alterations in both cellular abundance and BBB-related transcriptional signatures, subsequent analyses focused on this population. Differential expression analysis between ALS and control capillary endothelial cells was performed using Seurat’s FindMarkers function with a minimum expression frequency threshold of 20% (min.pct = 0.2). Given the reduced cell numbers following endothelial subtyping, a higher expression-frequency threshold was applied to minimize differential signals driven by genes expressed in only a small fraction of cells. In contrast, a moderately permissive fold-change threshold was used to retain genes showing consistent but potentially modest transcriptional changes within this closely related endothelial population. Genes exhibiting significant differential expression (adjusted *p*-value < 0.05 and log2 fold change > 0.25) were subsequently subjected to gene ontology (GO) enrichment analysis using clusterProfiler (v4.16.0) [70]. Human gene symbols were converted to Entrez identifiers using org.Hs.eg.db (v3.21.0), and enrichment analyses were performed for biological process (BP) terms. GO items with q-value (Benjamini-Hochberg) > 0.05 were filtered out.

### Gene regulatory network analysis in spinal cord capillary cells

To investigate transcriptional regulatory mechanisms underlying endothelial dysfunction in ALS, gene regulatory network (GRN) analysis was performed on spinal cord capillary endothelial cells using the pySCENIC (v0.12.1) [64]. Capillary endothelial nuclei identified from the spinal cord vascular atlas were exported as a gene expression matrix and analyzed using pySCENIC in three sequential steps: GRN inference, regulon identification, and regulon activity scoring. First, transcription factor (TF)–target gene co-expression networks were inferred using the GRNBoost2 algorithm implemented in pySCENIC. Second, candidate regulatory interactions were refined through cis-regulatory motif enrichment analysis using the pySCENIC ctx module. The hg38 cisTarget motif databases containing genomic regions located ±10 kb from transcription start sites were used to identify direct TF target genes and construct regulons. Dropout masking was enabled during motif enrichment analysis to improve robustness against sparse single-cell expression profiles. Third, regulon activity was quantified in individual nuclei using the AUCell algorithm, which calculates the enrichment of regulon target genes within the expressed gene set of each cell. The resulting regulon activity matrix was imported into Seurat as an independent assay for downstream analyses.

To identify disease-associated transcriptional regulators, regulon specificity scores (RSS) were calculated using the SCENIC package, comparing ALS and control capillary endothelial cells. Regulons were ranked according to their RSS values in each condition by the calcRSS function in R, and the highest-ranking ALS- and control-associated regulons were selected for visualization. Differences in regulon activity between ALS and control endothelial cells were assessed using two-sided Wilcoxon rank-sum tests. Regulon activity comparisons here between ALS and control cells were performed at the individual-cell level without explicit donor-level modeling.

### Quiescent spinal cord vascular data processing and correspondence analysis

To establish an atlas of quiescent vascular cells in the human spinal cord, snRNA-seq data from six neurologically healthy human spinal cord donors were downloaded from GEO (accession numbers: GSE190442) [72]. Raw count matrices and accompanying metadata were imported into Seurat and merged into a single object. Samples were integrated using Harmony-based batch correction implemented through Seurat v5 layer integration workflows. Briefly, gene expression data were normalized, highly variable genes were identified, scaled, and subjected to PCA. Batch effects among donors were corrected using Harmony, followed by nearest-neighbor graph construction, UMAP dimensionality reduction, and graph-based clustering. To generate a vascular-specific atlas, cells annotated as endothelial cells, pericytes, or meningeal populations in the original publication were extracted and reanalyzed. Vascular cell populations were identified according to canonical marker genes and manually annotated as arterial, capillary, and venous endothelial cells, pericytes, smooth muscle cells, meningeal fibroblasts, and two major perivascular fibroblast populations.

To evaluate the correspondence between vascular cell populations identified in ALS spinal cord samples and those present in healthy spinal cord tissue, the ALS vascular atlas (Dataset 1) and healthy vascular atlas (Dataset 2) were merged and analyzed using the MetaNeighbor (v1.28.0) [9]. First, the two datasets were merged into one object and the top 2000 highly variable genes (HVGs) were identified using Seurat’s FindVariableFeatures function. Then, the cell-type similarity was quantified using the unsupervised MetaNeighbor (MetaNeighborUS) algorithm based on these HVGs with default parameters. MetaNeighbor calculates pairwise area-under-the-receiver-operating-characteristic (AUROC) scores to assess the reproducibility and correspondence of cell identities across independent datasets.

### Immunofluorescence in *SOD1*^*G93A*^ transgenic and wild-type mice

#### Animals and CNS tissue collection

B6.Cg-Tg(SOD1*G93A)1Gur/J (*SOD1*^*G93A*^) mice, originally sourced from The Jackson Laboratory (JAX, #004435), were maintained on a C57BL/6 J background. The *Col1a1*-green fluorescence protein (GFP) reporter mice were obtained from Dr. Henrik Jessen Jürgensen at the University of Copenhagen. To obtain *SOD1*^*G93A*^ mice with fibroblast-specific GFP, *SOD1*^*G93A*^ male mice were crossbred with heterozygous *Col1a1*-GFP female mice. All procedures were approved by the Swedish National Board for Research Animal Ethics in accordance with European Community Council Directive 86/609/EEC and were conducted under ethical permit Dnr 23561-22 at the Department of Comparative Medicine, Karolinska University Hospital, Sweden.

Regarding tissue harvest for gene expression analysis, seven end-stage (around 20-week-old) *SOD1*^*G93A*^ mice and age-matched non-transgenic littermates of mixed sexes were used. Mice were euthanized by isoflurane overdose and transcardially perfused with 10 mL of ice-cold PBS. The vertebral column was opened using micro-scissors to expose the spinal cord. Tissue intended for RNA work was submerged in RNAlater solution (Invitrogen, #AM7020), snap-cryopreserved on dry ice, and stored at −80 °C until use.

GFP-positive mice with *SOD1* transgene at end-stage or age-matched non-*SOD1* control mice were used for histology analysis (n = 4–5, mixed sexes). Tissue designated for histology was post-fixed in 4% PFA for 4–6 h at 4 °C, cryopreserved through sequential overnight treatment in 20% and 30% sucrose solution, embedded in Tissue-Tek OCT Compound (Sakura, #4583), and preserved at −80 °C for long-term storage.

#### Immunofluorescence (IF)

Spinal cord cryosections (16 μm) were cut on a cryostat (Leica, CM1850) at −20 °C and mounted onto glass slides. Before staining, slides were equilibrated at room temperature for 30 min and rinsed in PBS (ThermoFisher, #18912014). Antigen retrieval was carried out with 0.025% trypsin–EDTA for 5 min, followed by a PBS wash. Sections were then blocked for 1 h at room temperature in 5% normal donkey serum containing 0.3% Triton X-100 in PBS. Sections were then incubated with rat anti-mouse laminin α1 antibody (kindly provided by Prof. Sorokin's lab[58]) for 36 h at 4 °C. After three PBS washes, laminin α1 immunoreactivity was detected using goat anti-rat Alexa Fluor 568 secondary antibody (Invitrogen, A11077, 1:600) for 1 h at room temperature. For mouse IgG staining, sections were incubated with donkey anti-mouse Alexa Fluor 647 secondary antibody (Invitrogen, A31571, 1:1000), and Alexa Fluor 488-conjugated anti-collagen IV antibody (SouthernBiotech, 1340-30, 1:400) was used to visualize all vessels. All sections were counterstained with DAPI (ThermoFisher, #62248, 1:10000) and cover slipped using antifade mount (Invitrogen, #P36980). Images were captured on a Zeiss LSM880 confocal microscope. The spatial distribution of LAMA1^high^ and LAMA1^low^ perivascular fibroblasts was qualitatively assessed based on their relative localization to the pial surface, blood vessels, and surrounding neural parenchyma.

### Human spinal cord and brain bulk RNA-seq data processing

To validate transcriptional alterations identified in vascular fibroblast populations, bulk RNA-seq datasets from human ALS spinal cord and cerebral cortex tissues were analyzed. Pan-fibroblast and subtype-specific marker genes, which are exclusively expressed in the fibroblast population compared to other cell types, were evaluated for differential expression between ALS and control samples.

#### For lumbar spinal cord bulk RNA-seq data

Processed gene expression count matrices from human lumbar spinal cord samples with de-identified metadata were obtained from Zenodo (https://doi.org/10.5281/zenodo.6385747) [25]. Samples annotated as ALS-frontotemporal dementia (ALS-FTD) were excluded from the ALS group. Expression levels of selected vascular fibroblast-associated genes, including *COL1A1*, *COL1A2*, *DCN*, *LUM*, *THBS2*, *CLDN1*, and *GADL1*, were extracted for downstream analyses. For each gene, expression distributions were compared between ALS and control samples using Wilcoxon rank-sum tests.

#### For cerebral cortex bulk RNA-seq analysis

Cortical bulk RNA-seq data were obtained from the NYGC ALS Consortium (GSE137810) [63]. Raw count matrices and associated sample metadata were downloaded and processed. Because sequencing was performed using two different platforms (GPL24676 and GPL24677), only samples generated using the GPL24676 platform were included to minimize technical batch effects and ensure availability of count data for downstream analyses. Samples originating from spinal cord tissue were excluded, and only ALS and neurologically normal control (CTL) cortical samples were retained. Gene-level count matrices were merged across samples and matched to corresponding clinical metadata. After this, the raw gene counts were normalized using the size-factor normalization implemented in DESeq2 (v1.48.2) to account for differences in library size across samples. Size factors were estimated using the estimateSizeFactors function, and normalized counts were extracted for subsequent expression comparisons. Expression levels of the same fibroblast-associated genes examined in the spinal cord dataset were extracted and compared between ALS and control cortical tissues. Statistical significance was assessed using Wilcoxon rank-sum tests.

### Quantitative real-time PCR of fibroblast markers in *SOD1*^*G93A*^ and wild-type mice

Total RNA was isolated using the RNeasy Lipid Tissue Mini Kit (Qiagen, #74804) for mouse CNS tissue according to the manufacturer's standard protocols, with on-column genomic DNA removal performed using the RNase-Free DNase Set (Qiagen, #79254). Following quantification, 500 ng of RNA was reverse-transcribed using a cDNA synthesis kit (Bio-Rad, #1708891). Gene transcripts were quantified by SYBR Green qPCR (Bio-Rad, #1708886) according to the manufacturer's instructions in a 384-well plate run in a Bio-Rad CFX384 Touch Real-Time PCR Detection System. The cycling protocol consisted of 95 °C for 30 s as well as 40 cycles of 95 °C for 5 s followed by 60 °C for 30 s, with a melt curve analysis performed after amplification. Gene expression was measured in seven biological replicates per group, each analyzed in triplicate. Relative expression was determined by the 2^−ΔΔCt^ method, normalized to the mouse *Rpl19* gene, and all murine primers were verified by melt-curve analysis. Primer and probe sequences are provided in Supplementary Table 3.

### Cell–cell communication analysis in vascular cell types

To investigate intercellular communication within the spinal cord vascular niche and determine how signaling networks are altered in ALS, cell–cell communication analyses were performed using the CellChat (v2.2.0) [28]. Meningeal fibroblasts were excluded from the spinal cord vascular atlas for subsequent communication analyses because of their limited representation in control samples. The dataset was subsequently divided into ALS and control groups for independent network inference. For each condition, normalized gene expression matrices were used to construct CellChat objects with cell-type annotations specified as communication groups. The human CellChat ligand–receptor database was employed as the reference interaction resource. CellChat analyses were performed following the standard workflow with default parameters. To compare communication networks between ALS and control samples, condition-specific CellChat objects were merged into a unified analysis framework. Differential pathway activity between ALS and control networks was evaluated using CellChat’s pathway-ranking framework. Signaling pathways exhibiting significant differences in communication strength were identified using statistical comparisons with a significance threshold of *p* < 0.01. Particular attention was given to extracellular matrix-related signaling pathways, including LAMININ, COLLAGEN, FN1, AGRN, and CXCL signaling.

### Fibroblast state module scoring

To characterize functional state transitions within vascular fibroblasts, downstream analyses focused on P. Fibroblast type2, a fibroblast population exhibiting marked disease-associated activation in ALS spinal cord samples. Cells belonging to this population were extracted from the integrated vascular atlas and further subdivided into two transcriptionally distinct subclusters based on unsupervised clustering (c1 and c2). In detail, we ran the dimension reduction for the fibroblast population as before but increased the clustering resolution to 0.5. Following the removal of outlier cells identified in UMAP space, cellular compositions of fibroblast subclusters were quantified across disease conditions. To investigate fibroblast activation states, transcriptional state signatures were obtained from a recently published pan-fibroblast perturbation study [59]. Four conserved fibroblast programs were evaluated: myofibroblast, inflammatory, antigen presentation, and universal fibroblast states. Corresponding gene sets were imported and scored at the single-cell level using the AddModuleScore function. Statistical significance was assessed using two-sided Wilcoxon rank-sum tests.

### Pseudo-time trajectory in P. Fibroblast type2

To investigate dynamic transcriptional transitions within P. Fibroblast type2 pseudotime trajectory analysis was performed using Monocle3 (v1.4.27) [4]. A CellDataSet object was constructed from raw UMI count matrices. Data preprocessing included dimensionality reduction using principal component analysis, followed by donor-level batch correction using the align_cds function to minimize inter-individual variability. Low-dimensional embeddings were replaced with the corresponding Seurat UMAP coordinates to maintain consistency between clustering and trajectory analyses. Principal trajectories were learned using Monocle3’s graph-learning algorithm. Cellular pseudotime values were then inferred by ordering cells along the learned trajectory graph. To identify genes associated with fibroblast state progression, differential expression along pseudotime was evaluated using Monocle3’s graph-based Moran’s I test (graph_test). Genes exhibiting the strongest pseudotime-dependent expression patterns were ranked according to Moran’s I statistics and visualized across the inferred trajectory.

## Results

### Human spinal cord and brain vascular cell map in ALS

**Fig. 1:**
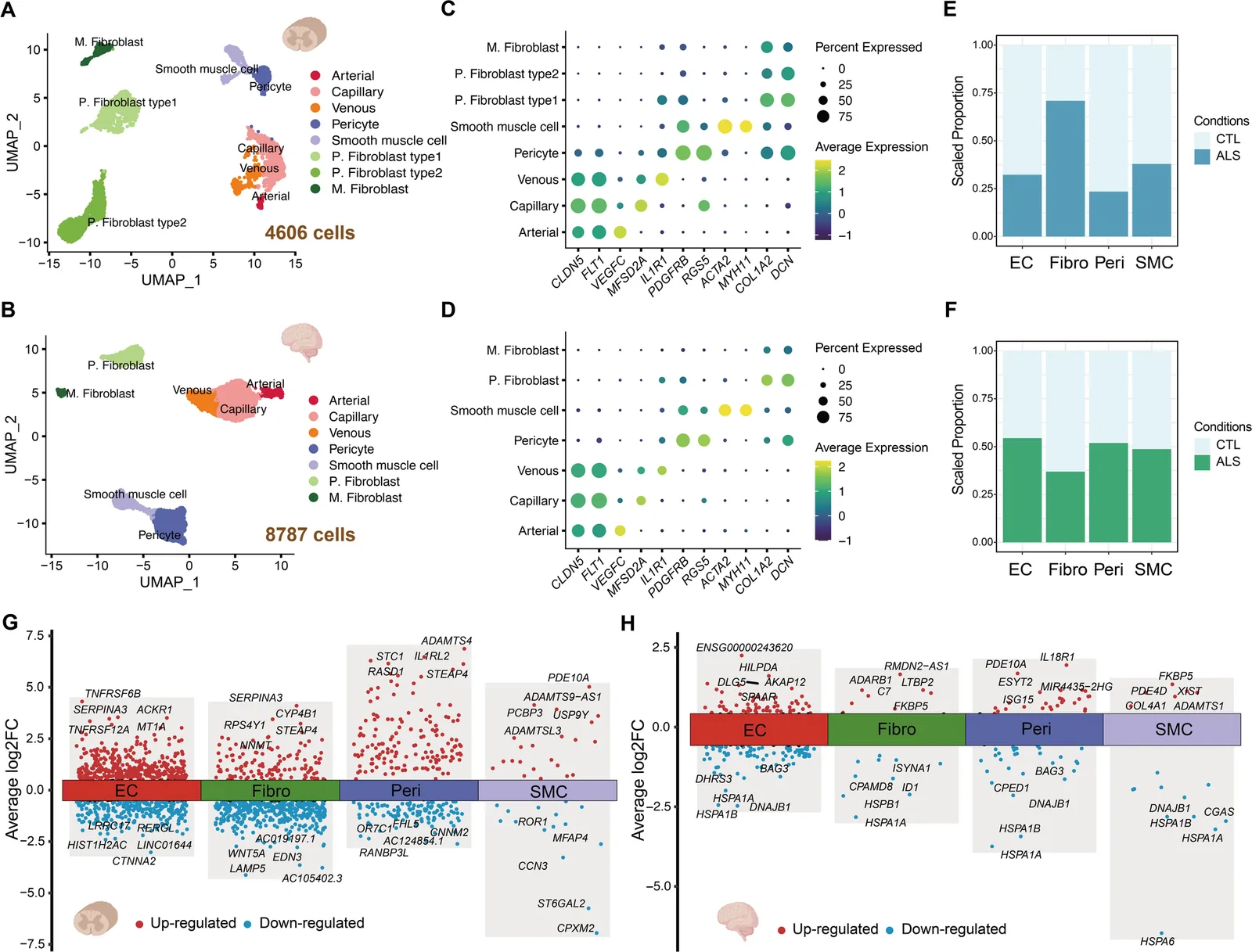
Human spinal cord and brain vascular cell map in ALS. **A**, **B**. Uniform manifold approximation and projection (UMAP) plot showing the vascular cell composition in human spinal cord (**A**) and cortex (**B**). P. Fibroblast, pia/perivascular fibroblast. M. Fibroblast, meningeal fibroblast. **C**, **D**. Dot plot showing the expression of canonical marker genes for vascular cell types in spinal cord (**C**) and cortex (**D**). **E**, **F**. Cell proportions of ALS and healthy control for each major cell class in spinal cord (**E**) and cortex (**F**). Cell numbers of all cell classes from each group were normalized to their total cell amount. CTL, healthy control group. EC, endothelial cell. Fibro, fibroblast. Peri, pericyte. SMC, smooth muscle cell. **G**, **H**. Scatter plot showing the differentially expressed genes between ALS and healthy controls in spinal cord (**G**) and cortex (**H**). Up-regulated genes: adjusted *p*-value < 0.05 and log2(fold change) > 0.5. Down-regulated genes: adjusted *p*-value < 0.05 and log2(fold change) < − 0.5. The top 5 genes are annotated

Given the emerging evidence implicating neurovascular dysfunction in ALS, we sought to structurally characterize its impact on different vascular cell populations in the human CNS. Although recent multi-omics studies, including snRNA-seq, have reported substantial cellular alterations in ALS, a comprehensive evaluation of vascular cell populations remains incomplete. To address this, we curated multiple snRNA-seq datasets derived from the human motor cortex and spinal cord of ALS patients and controls to construct a primary vascular cell atlas in ALS (Fig. 1A, B, S1A, and S1B).

After stringent quality control and batch effect correction, we obtained 4606 cells (including 3303 for ALS and 1303 for healthy controls) from the spinal cord (SC) and 8787 cells (including 7532 for ALS and 1255 for healthy controls) from the cortex (CTX; predominantly motor cortex) (Fig. 1A, B, S1C, and S1D). Based on well-established marker genes, we annotated eight cell types spanning four major vascular classes: endothelial cells (arterial, capillary, and venous), fibroblasts (meningeal fibroblasts and pia/perivascular fibroblast types 1 and 2), pericytes, and smooth muscle cells (Fig. 1C, D). Of note, perivascular macrophages (PVMs) were excluded from this study because of their distinct functional characteristics and the current difficulty in accurately distinguishing them from circulating macrophages in single-cell omics data.

We next examined changes in cell-type composition between ALS and control samples. Notably, the spinal cord exhibited more pronounced alterations compared to the cortex, where cell-type proportions remained relatively stable between conditions (Fig. 1E, F), indicating region-specific vascular alterations in ALS. In the spinal cord, fibroblast populations were markedly increased in ALS, consistent with our previous findings [40]. In contrast, other cell types, like pericytes, were relatively reduced in ALS as reported before [69]. This regional difference was further supported by our differential gene expression analysis (Table S1). The spinal cord exhibited substantially more DEGs across all four vascular classes compared with the cortex (Fig. 1G, H). In the spinal cord, endothelial cells showed the highest number of upregulated DEGs, whereas fibroblasts exhibited the largest number of downregulated genes (Fig. S1E and S1F). For example, tumor necrosis factor (TNF) receptor genes, such as *TNFRSF6B*, were significantly upregulated in ALS endothelial cells from the spinal cord, which have been implicated in BBB disruption and neuroinflammation [38, 57]. The atypical chemokine receptor (ACKR1), which facilitates immune cell trafficking across the BBB, was also increased in ALS spinal cord endothelial cells [18, 41]. In addition, *SERPINA3*, a gene broadly associated with neuroinflammation in AD [76], was found to be significantly upregulated in ALS vascular cells as well. In contrast, the cortex displayed relatively modest transcriptional changes, with endothelial cells being the primary cell type exhibiting differential expression between ALS and controls (Fig. 1H). Genes such as *HILPDA*, *DLG5*, and *AKAP12* were upregulated in ALS cortex endothelial cells and have been linked to hypoxia responses and vascular homeostasis [6, 11, 33].

Although the number of identified DEGs may be influenced by cell abundance across vascular classes, these results nonetheless highlighted prominent transcriptional alterations in endothelial cells and fibroblasts in ALS. Strikingly, only a small fraction of DEGs were shared between the spinal cord and cortex across corresponding vascular cell types (Fig. S1E and S1F). Even in endothelial cells, which showed the greatest overlap, only eight DEGs were uniquely shared (out of 11 overlapping genes, three of which were also shared with other cell types) (Fig. S1G and S1H). This limited overlap indicates that ALS induces largely region-specific transcriptional programs in the CNS vasculature.

Here, we established a curated vascular cell atlas from the human spinal cord and cortex datasets in ALS. Based on that, our analysis revealed substantial vascular perturbations at both the cellular and transcriptional levels, with more pronounced changes in the spinal cord compared to the cortex. Among vascular cell types, endothelial cells and fibroblasts appeared to be the most transcriptionally affected in ALS.

### Endothelial alterations reveal impaired BBB-associated transcriptional signatures in the ALS spinal cord

Endothelial cells constitute the primary structural component of the vasculature and represent one of the most abundant cell types within the neurovascular unit. Their dysfunction has been increasingly recognized as an early and critical event across neurodegenerative disorders [61]. Consistent with this, our analysis identified substantial transcriptional alterations in endothelial cells in ALS (Fig. 1G, H). We therefore next focused on endothelial subtypes to further dissect their molecular changes and potential functional consequences in disease pathology.

**Fig. 2:**
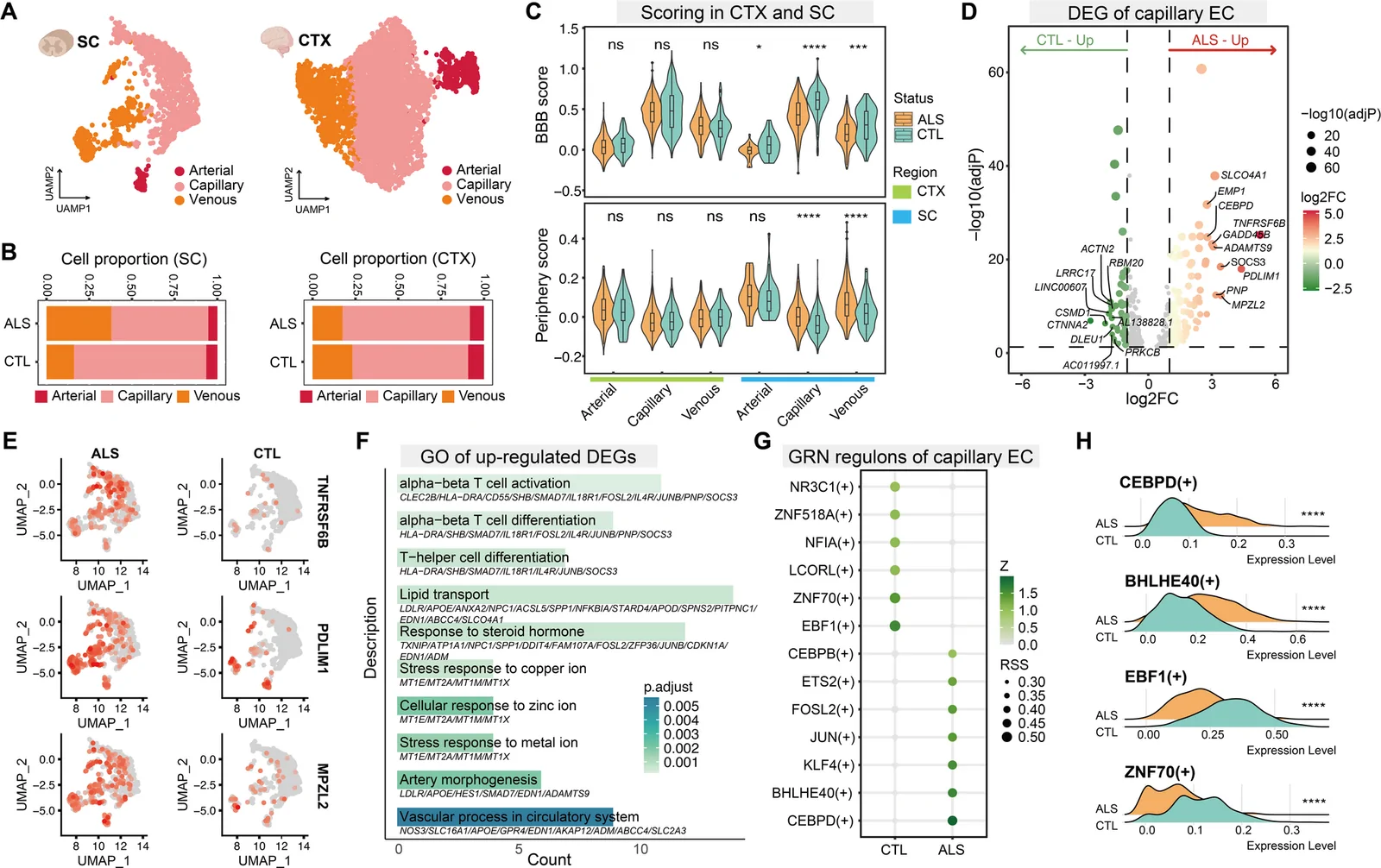
Transcriptional alterations in capillary endothelial cells in ALS. **A**. The zonation of endothelial cells in the spinal cord (Left) and cortex (Right) at the transcriptional level. **B**. Difference in the proportions of three types of endothelial cells in ALS and CTL from the spinal cord (Left) and cortex (Right). **C**. Assessment of BBB (Up) and peripheral scores (Down) across vessel types in ALS vs Control. *, *p* < 0.05. ***, *p* < 0.001. ****, *p* < 0.0001. ns, not significant. Wilcoxon rank-sum test. **D**. Volcano plot of DEGs from capillary endothelial cells in the spinal cord. − log_10_(adjP), − log_10_(adjusted *p* value). Log_2_FC, log_2_(fold change). **E**. Feature plot showing the different expression of selected DEGs in ALS endothelial cells vs. control. **F**. Gene ontology (GO) enrichment analysis of up-regulated DEGs in spinal cord capillary cells. **G**. Dot plot showing condition-specific gene regulatory networks (GRNs) in spinal cord capillary cells. **H**. Showcase of the regulon activity level in spinal cord capillary cells between ALS patients and healthy controls. ****, *p* < 0.0001. Wilcoxon rank-sum test

With differentiated transcriptional characteristics, we reconstructed endothelial cell zonation, identifying arterial, capillary, and venous populations (Fig. 2A). In line with the general compositional differences, the spinal cord exhibited more pronounced alterations than the cortex, including a marked reduction in capillary endothelial cell proportions, consistent with our previous findings of reduced capillary density [8, 34] (Fig. 2B). Previous studies of healthy and diseased cerebral vasculature have shown that BBB disruption is often accompanied by loss of canonical BBB signatures and a shift of endothelial characteristics towards a peripheral tissue phenotype [47, 66]. For the cortex, we did not observe significant differences in either BBB or peripheral signature scores across arterial, capillary, or venous compartments between ALS and control groups (Fig. 2C), suggesting relatively preserved vascular integrity. In contrast, the spinal cord displayed a significant decrease in BBB-associated signatures together with a concomitant increase in peripheral signatures across vascular subtypes (Fig. 2C). Of note, these changes were particularly pronounced in capillary endothelial cells, indicating a substantial transcriptional shift from BBB-associated toward peripheral-like endothelial characteristics in the ALS spinal cord [68]. To further assess whether these transcriptional alterations were accompanied by vascular barrier disruption, we performed immunofluorescence staining for collagen IV and IgG in the spinal cord of an ALS mouse model. Increased extravascular IgG accumulation surrounding collagen IV-positive vessels was observed in ALS mice, providing complementary histological evidence of BSCB leakage (Fig. S2).

Given the prominent alterations in capillary endothelial cells in the spinal cord, we next examined their transcriptional changes in detail (Fig. 2D and Table S2). Despite the reduced abundance of capillary cells, the remaining endothelial cells exhibited increased expression of *TNFRSF6B* (Fig. 2E), suggesting activation of anti-apoptotic and immune-modulatory pathways in response to inflammatory stress [53]. In parallel, migration- and adhesion-related genes, including *PDLIM1* and *MPZL2*, were upregulated in ALS, indicative of cytoskeletal remodeling and functional reprogramming of endothelial cells, which may affect BSCB integrity and vascular permeability (Fig. 2E). Subsequently, GO enrichment analysis of upregulated genes in ALS capillary endothelial cells revealed significant enrichment of pathways related to T cell activation (Fig. 2F). While T lymphocyte infiltration in the ALS spinal cord has been well documented [23, 55], these results further suggest altered immune-related biological processes in the vasculature under disease conditions in human samples. In addition, pathways associated with lipid metabolism dysregulation and metal ion stress were also enriched, which are recognized hallmarks of ALS pathology (Fig. 2F). Moreover, GO terms related to vascular remodeling and endothelial activation were also enriched, supporting the notion of functional reprogramming in ALS capillary cells (Fig. 2F).

To further characterize these changes, gene regulatory network analysis revealed distinct shifts in transcriptional regulation in ALS capillary endothelial cells (Fig. 2G). For instance, CEBPD and BHLHE40 regulons, both established stress- and inflammation-responsive factors, showed increased activity in ALS, whereas EBF1 and ZNF70 were suppressed, potentially reflecting a loss of homeostatic vascular regulatory programs [30, 45] (Fig. 2H).

Although alterations of the BBB/BSCB have been reported in several ALS mouse models [5, 15, 50, 75], our analysis adds new evidence from the estimation of BBB/BSCB-associated transcriptional changes in human tissues, highlighting a pronounced endothelial transformation in the spinal cord but not in the cortex. This discrepancy between the spinal cord and cortex also underscores the region-specific pathology of ALS. Moreover, dysregulated pathways and regulatory networks suggest that capillary endothelial cells in the ALS spinal cord undergo coordinated transcriptional and regulatory reprogramming towards a stressed and permeable state.

### Identification of a distinct perivascular fibroblast subtype in the human spinal cord

**Fig. 3:**
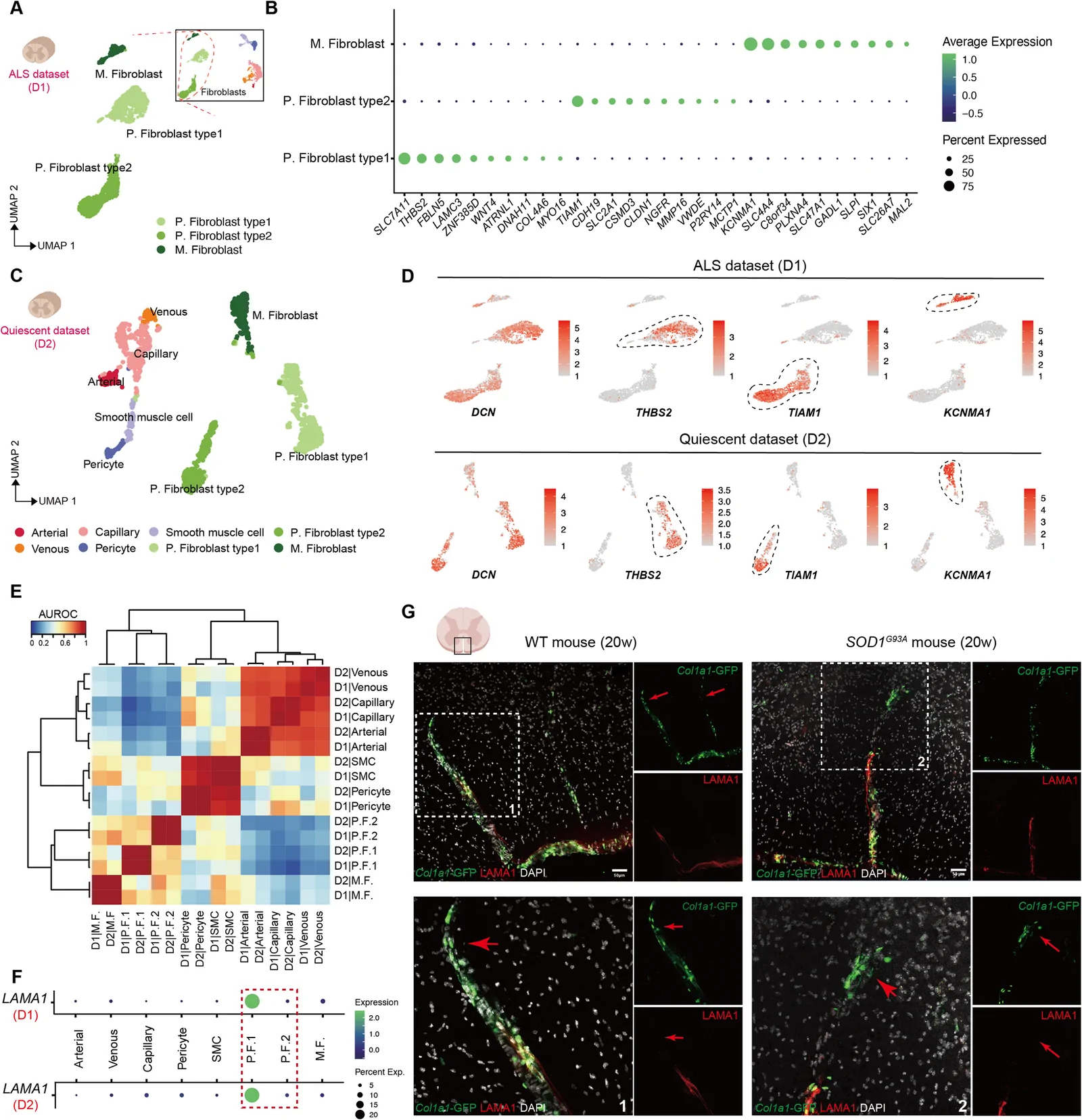
Spatially segregated perivascular fibroblast populations in the human spinal cord. **A** UMAP plot of fibroblast types in the ALS spinal cord.** B**. Dot plot showing the top differentially expressed genes among three fibroblast populations. **C**. UMAP plot representing the vascular map from the healthy human spinal cord. **D**. Pan-fibroblast and type-specific marker expression in two datasets. **E**. Corresponding relationship between two independent datasets by MetaNeighbor. P.F.1, pia/perivascular fibroblast type 1. P.F.2, pia/perivascular fibroblast type 2. M.F., Meningeal fibroblast. **F**. Dot plot showing LAMA1 expression among different vascular populations in two datasets. **G**. Immunofluorescence of LAMA1 in spinal cords from *SOD1*^*G93A*^ transgenic and wild-type mice, both of which are crossed with the *Col1a1*-GFP reporter. The red arrows here indicate the *Col1a1*-GFP+ LAMA1- fibroblasts. Scale bar: 50 µm

Fibroblasts are increasingly recognized as important regulators of CNS homeostasis and pathology [12]. However, their heterogeneity under physiological conditions remains incompletely characterized, particularly in the human spinal cord. To systematically define fibroblast diversity, we re-clustered fibroblast populations from the human spinal cord in our working dataset and identified three transcriptionally distinct fibroblast populations (Fig. 3A).

One cluster corresponded to canonical meningeal fibroblasts (M. Fibroblast), characterized by expression of established markers including *KCNMA1* and *SLC4A4* [17, 73] (Fig. 3B). Additional genes enriched in this population included *SLC47A1*, *GADL1*, *SIX1*, etc. In contrast, pia/perivascular fibroblasts segregated into two discrete subpopulations, which we termed P. Fibroblast type1 and P. Fibroblast type2, respectively (Fig. 3A). P. Fibroblast type1 preferentially expressed *SLC7A11*, *THBS2*, *FBLN5*, and *LAMC3*, whereas P. Fibroblast type2 was distinguished by enrichment of *TIAM1* and *CDH19*. To our knowledge, this subdivision of spinal cord pia/perivascular fibroblast subtypes has not been clearly described in previous human CNS vascular studies.

To further confirm the fibroblast heterogeneity revealed in the human spinal cord data we used here (D1), we further included another independent dataset (D2) from neurologically normal donors [72] (Fig. 3C and S3A). After a similar unsupervised clustering workflow, the cell composition of the quiescent vascular dataset closely resembled the result from D1. Marker gene expression patterns were also highly consistent between datasets (Fig. 3D). To measure the correspondence of cell types across two datasets quantitatively, we applied a neighbor-voting approach based on cell–cell similarities to all vascular cells [9]. All major vascular classes show exclusive similarity across datasets, and the fibroblast subtypes presented strong one-to-one correspondence as well, which further demonstrated the heterogeneity of P. Fibroblast in human spinal cord and the robustness of our methodology (Fig. 3E). We next examined whether this fibroblast architecture was conserved across species using a mouse spinal cord dataset [71]. Notably, transcriptionally analogous fibroblast populations were also detected in the mouse spinal cord, including a population resembling human P. Fibroblast type2 (Fig. S3B), suggesting that the major organizational framework of spinal cord fibroblasts is conserved among mammals.

Functionally, DEGs from all three types of fibroblasts in human spinal cord (D1) are enriched in the extracellular matrix (ECM) organization and encapsulating structure formation (Fig. S3C), consistent with their canonical role in maintaining CNS connective tissues. Compared with meningeal fibroblasts, the P. Fibroblast type1 showed more enriched pathways associated with vascular transport, cell–matrix adhesion, and blood coagulation. These findings are consistent with the closer anatomical proximity of P. Fibroblasts to the vasculature compared with meningeal fibroblasts, further supporting their specialized role within the neurovascular niche. However, we found P. Fibroblast type2 showed a more sensitive response to lipopolysaccharide (LPS) and corticosteroid, indicating its association with multiple biological programs in the CNS (Fig. S3C). Interestingly, P. Fibroblast type2 additionally exhibited enrichment for axonogenesis-related programs (Fig. S3C). This feature resembled a previously reported nerve-associated fibroblast (NAF) population in the peripheral nervous system, which secretes neurotrophic factors to promote axon growth [22, 39]. Previous work has reported major NAF markers, including *NGFR*, *EBF2*, *SCN7A*, and *CLDN1* [14]. Here, we also found that those NAF markers were selectively expressed in the P. Fibroblast type2 population within two independent human datasets (Fig. S3D). Together, these findings indicate that P. Fibroblast type2 represents a molecularly distinct perivascular fibroblast population with transcriptional features shared with peripheral nerve-associated fibroblasts.

Laminin subunit alpha-1 (LAMA1) is a canonical marker that has been broadly used for the identification of pia/perivascular fibroblasts in past mammalian CNS studies [51]. Here, in the human datasets, we found that P. Fibroblast type1 highly expressed *LAMA1*, which is aligned with the previously identified typical pia/perivascular fibroblasts, whereas the P. Fibroblast type2 presented a feature of low *LAMA1* expression (Fig. 3F), suggesting distinct spatial identities. To further substantiate the existence of two types of P. Fibroblast in the spinal cord and clarify their anatomical distribution, we performed immunofluorescence (IF) in both wild-type and *SOD1*^*G93A*^ transgenic mouse spinal cords. Consistent with this prediction, we discerned a specialized zonation pattern of fibroblast populations along the vascular wall in the mouse spinal cords (Fig. 3G). The LAMA1^high^ fibroblasts were predominantly distributed in the pia and superficial perivascular regions, corresponding to the canonical pia/perivascular fibroblast population described previously. In contrast, the LAMA1^low^ fibroblasts were preferentially located in deeper perivascular regions adjacent to arterioles and the parenchyma, in accord with their multi-functional assumption and direct interaction with nervous system. This spatial organization was consistently observed across two mouse strains (wild-type and *SOD1*^*G93A*^) among biological replicates (Fig. 3G and S3E).

Collectively, these analyses identify a previously unrecognized perivascular fibroblast subtype in the human spinal cord. Unlike the classical *LAMA1*^high^ pia/perivascular fibroblasts, this population exhibits distinct molecular signatures, neurovascular-associated functions, and a deeper perivascular localization. Notably, a comparable degree of pia/perivascular fibroblast heterogeneity was not evident in our human brain dataset (Fig. 1A). Likewise, most previously reported human brain vascular atlases do not report a clear segregation of perivascular fibroblast subpopulations [17, 51, 54, 67, 73]. Although regional sampling bias cannot be excluded, these findings suggest that fibroblast specialization may be more pronounced in the spinal cord than in the brain. Together, our results refine the cellular taxonomy of CNS fibroblasts and reveal previously unrecognized spatial and functional heterogeneity within the spinal cord neurovascular niche.

### Fibroblast signature elevation and activation trajectory in ALS spinal cord

**Fig. 4:**
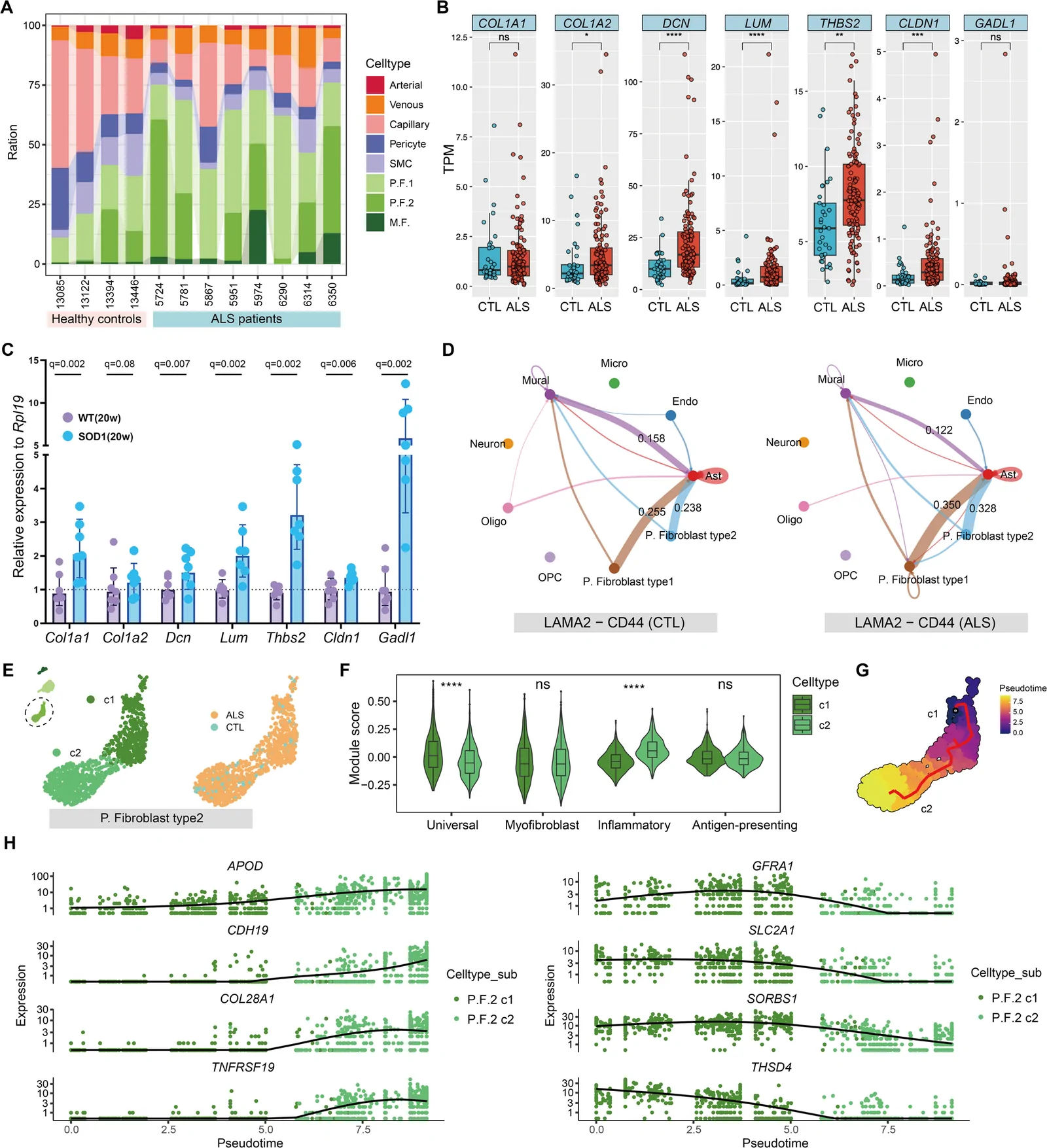
Disease-associated remodeling of perivascular fibroblast type 2 in ALS. **A** Vascular cell proportion of spinal cords from ALS patients and healthy controls. **B**. Selective pan-fibroblast and type-specific marker expression in spinal cord bulk RNA-seq data. TPM, transcripts per million. *, *p*<0.05. **, *p*<0.01. ***, *p*<0.001. ****, *p*<0.0001. ns, not significant. Wilcoxon rank-sum tests. **C**. qPCR of pan-fibroblast and type-specific markers in *SOD1*^*G93A*^ mice (n=7) and wild-type mice (n=7). q, Benjamini-Hochberg (BH) procedure. **D**. Interaction among major cell types in the healthy control (Left) and ALS (Right) groups through the LAMA2-CD44 axis. A connected edge between two cell types indicates communication probability with a *p*-value < 0.01. **E**. Sub-clustering of P. Fibroblast type2. **F**. Module scores of four typical fibroblast states on subtypes of P. Fibroblast type2. **G**. Pseudotime trajectory of P. Fibroblast type2 from c1 to c2. H. Top differently expressed genes along the trajectory from c1 to c2

Based on the pronounced fibroblast transcriptional heterogeneity and alterations in ALS (Figs. 1E, G, and 3A), we next sought to characterize fibroblast-specific changes in ALS in greater detail. Consistent with our previous observations of perivascular fibroblast activation during disease progression [40], we observed a general elevation of fibroblast populations in ALS spinal cords despite considerable inter-individual variability within the dataset (Fig. 4A). Notably, the increase was primarily attributable to perivascular fibroblast populations, suggesting their susceptibility to disease-associated remodeling.

To validate the expansion of fibroblasts in ALS, we examined the expression of pan-fibroblast markers (*COL1A1*, *COL1A2*, *DCN*, and *LUM*) together with subtype-specific markers (*THBS2* for P. Fibroblast type1, *CLDN1* for P. Fibroblast type2, and *GADL1* for M. Fibroblast) in an independent bulk RNA-sequencing cohort of human lumbar spinal cord tissues [25]. These marker genes were selected based on their selective enrichment within fibroblast populations compared to other major cell types (Fig. S4A and S4B). Most fibroblast markers were significantly elevated in ALS patients compared with healthy controls (Fig. 4B). Similar findings were observed in the spinal cord tissues from the end-stage *SOD1*^*G93A*^ transgenic mice (Fig. 4C and Table S3). Together, these results demonstrated a robust increase in fibroblast-specific gene expression in ALS across both human and mouse datasets. Of note, while P. Fibroblast markers were significantly dysregulated in both human ALS bulk data and mouse models, M. Fibroblast markers showed relatively variable results across experiments (significantly upregulated in mouse models but not reaching statistical significance in human ALS bulk data), which could be caused by the bias of meningeal fragment representation during the spinal cord sampling and processing (Fig. 2B, C). Contrary to the spinal cords, comparable alterations of fibroblast markers were not observed in ALS cortical bulk RNA-sequencing data [63] (Fig. S4C). This regional difference suggested that fibroblast activation may be more pronounced in the spinal cord, where neurovascular disturbances are substantially greater than those observed in the cortex.

To determine whether fibroblast activation was accompanied by remodeling of the neurovascular niche, we next performed cell–cell communication analysis on all major cell types from human spinal cord. Compared with healthy controls, ALS samples exhibited enhanced signaling through several inflammation- and remodeling-associated pathways, including VISFATIN, ADGRE, and SPP1 (Fig. S4D). Notably, distinct communication patterns were observed between the two perivascular fibroblast populations and major vascular cell types (Fig. S4E). Among these interactions, FN1-mediated signaling was strengthened in P. Fibroblast type2. As for the vascular ECM, the signaling mediated by COL4 family members was reduced in P. fibroblast type2, while COL9A3-related pathways were stronger in P. fibroblast type2 (Fig. S4E). Particularly, we found that the interactions from two types of perivascular fibroblasts to astrocytes through the LAMA2-CD44 axis were significantly elevated in the ALS group (Fig. 4D), indicating systematic extracellular matrix remodeling in ALS vasculature from fibroblast to astrocyte endfeet.

Given the pronounced alterations observed in P. Fibroblast type2, we next explored its internal heterogeneity through sub-clustering analysis. Two transcriptionally distinct states, designated c1 and c2, were identified (Fig. 4E). To characterize their functional identities, we projected a recently established fibroblast-state gene program derived from CRISPRa Perturb-seq studies [59]. Compared with c1, c2 displayed a significantly elevated inflammatory signature accompanied by a reduction in the universal fibroblast program (Fig. 4F). In contrast, no significant differences were observed in myofibroblast-associated or antigen-presenting programs between the two states. These findings suggest that c2 represents a more activated inflammatory state of P. Fibroblast type2.

Following the indication of a more activated state in c2, we constructed a pseudo-time trajectory to denote the transformation from c1 to c2 (Fig. 4G). Along this trajectory, several inflammation- and matrix remodeling-associated genes, including *APOD*, *CDH19*, *COL28A1*, and *TNFRSF19*, showed progressively increased expression, whereas genes associated with homeostatic functions, such as *GFRA1*, *SLC2A1*, *SORBS1*, and *THSD4*, gradually declined (Fig. 4H). These coordinated transcriptional changes support a transition from a homeostatic fibroblast state toward an inflammatory and remodeling-associated state during ALS progression.

Collectively, these findings demonstrate that fibroblasts are expanded and transcriptionally activated in the human ALS spinal cord. Among the identified fibroblast populations, P. Fibroblast type2 appears particularly responsive to disease-associated remodeling, exhibiting altered neurovascular interactions and a transition toward an inflammatory state. These observations further implicate perivascular fibroblasts as active participants in neurovascular dysfunction during ALS pathogenesis.

## Discussion

Increasing evidence suggests that disruption of BBB and BSCB integrity is a fundamental feature of numerous neurological disorders [60, 61]. Once regarded as a secondary consequence of neurodegeneration, vascular dysfunction is now increasingly recognized as an active contributor to disease initiation and progression [60, 75]. In ALS, pathological alterations of the neurovascular unit have been reported in both patients and experimental models [7, 24, 27, 40], yet a comprehensive characterization of vascular cell populations in the human central nervous system has remained lacking. By curating multiple human single-nucleus transcriptomic datasets, our study establishes a vascular-focused atlas of the ALS spinal cord and cortex and enables direct comparison of disease-associated alterations across vascular cell types and anatomical regions. This analysis reveals a pronounced regional divergence, with substantially greater vascular perturbations in the spinal cord than in the cortex and particularly prominent transcriptional alterations in endothelial cells and fibroblasts.

Endothelial cells constitute the structural foundation of the BBB and BSCB and are therefore central to maintaining CNS homeostasis. Previous studies in *SOD1*, *TARDBP*, and *C9orf72*-based animal models have demonstrated endothelial degeneration, barrier leakage, and impaired vascular perfusion during ALS progression [5, 15, 34, 50, 75]. Here, in our human transcriptomic analysis, spinal cord endothelial cells exhibited reduced BBB-associated transcriptional signatures together with increased peripheral endothelial signatures, whereas comparable changes were not evident in the cortex. Notably, capillary endothelial cells appeared particularly vulnerable, displaying activation of inflammatory, immune-response, and stress-associated pathways. Given the central role of capillary endothelial cells in regulating molecular exchange between the circulation and neural tissue, their pronounced transcriptional alterations identify this population as a potentially important component of ALS-associated neurovascular pathology.

Beyond endothelial alterations, our study provides a refined view of fibroblast heterogeneity within the human spinal cord. Although CNS fibroblasts have attracted increasing attention in recent years, particularly in injury and neuroinflammatory conditions, their diversity under physiological conditions remains incompletely understood [12]. Here, we identified three major fibroblast populations in the human spinal cord and further characterized a previously underappreciated *LAMA1*^low^ perivascular fibroblast population (P. Fibroblast type2). This population exhibited a transcriptional profile distinct from the classic *LAMA1*^high^ perivascular fibroblast population and expressed several markers previously associated with peripheral nerve-associated fibroblasts. Immunofluorescence on mouse spinal cords further revealed an apparent spatial distinction between *LAMA1*^high^ and *LAMA1*^low^ fibroblasts, with the former observed predominantly along the pia and superficial perivascular regions and the latter around deeper parenchymal vessels. These observations suggest that perivascular fibroblasts may be more heterogeneous than previously appreciated and raise the possibility that specialized fibroblast populations contribute differentially to neurovascular homeostasis within the CNS.

Importantly, fibroblast populations were consistently expanded in ALS spinal cord tissues across single-cell, bulk transcriptomic, and experimental validation datasets. Among the identified fibroblast populations, P. Fibroblast type2 displayed the most prominent disease-associated alterations. Sub-clustering and pseudotime analyses suggested a transition from a homeostatic state toward an inflammatory and remodeling-associated state within this population. While the precise functional consequences of this transition remain to be determined, these findings indicate that perivascular fibroblasts are not merely passive structural components of the neurovascular unit but may actively participate in vascular remodeling during ALS progression.

Several limitations should be considered when interpreting our findings. First, vascular cells represent a relatively small fraction of total CNS cells, and no vascular enrichment strategy was applied prior to sequencing in the datasets analyzed here. Consequently, certain vascular populations, particularly in cortical samples, were represented by limited cell numbers. In addition, differential expression analyses were performed at the individual-cell level in this study, while donor-level pseudoreplication analyses were limited by the small number of control spinal cord samples and the sparse and uneven representation of individual vascular subtypes across donors. Therefore, larger cohorts with more balanced vascular cell representation will be required to further validate these transcriptional changes at the donor level. Second, the number of neurologically normal human spinal cord samples available for comparison remains relatively small, which may influence estimates of cell-type abundance. Third, owing to limited sample size, ALS cases with different genetic backgrounds and clinical subtypes were analyzed together. Given the substantial heterogeneity of ALS, future studies incorporating larger cohorts and stratified analyses will be required to determine whether distinct vascular alterations are associated with specific molecular or clinical subgroups. Finally, several biological processes and intercellular interactions identified in this study were inferred from transcriptomic signatures, pathway enrichment, GRN analysis, pseudotime reconstruction, and computational cell–cell communication analysis. Although selected findings were supported by independent transcriptomic datasets and experimental validation, direct functional studies will be necessary to determine their biological consequences and roles in ALS progression.

## Conclusion

In summary, our integrated vascular atlas provides a region-resolved view of neurovascular alterations in human ALS and reveals substantially greater vascular perturbation in the spinal cord than in the cortex. By resolving endothelial and fibroblast heterogeneity, we identify a prominent disease-associated transcriptional shift in spinal cord endothelial cells and characterize a distinct *LAMA1*^low^ perivascular fibroblast population supported by cross-dataset and experimental evidence. Together, these findings expand the neurovascular framework of ALS pathology and identify regional endothelial and perivascular fibroblast remodeling as prominent features of human ALS. This framework provides a foundation for future mechanistic studies to determine how these vascular-associated alterations contribute to ALS progression.

## Supplementary Information

Below is the link to the electronic supplementary material.

Supplementary Figures

Supplementary Table 1

Supplementary Table 2

Supplementary Table 3

## Data Availability

All transcriptomic data used in this study are publicly accessible and the respective sources are described in the Materials and Methods paragraph. Codes used in the data analysis and visualization have been deposited in an open-access GitHub repository (https://github.com/Coleliao/VascularALS).
