# Supplementary material for: Vascular profiling of the spinal cord and cortex reveals region-specific endothelial dysfunction and fibroblast remodeling in human ALS
Source: Acta Neuropathol Commun. 2026 Oct 10;14:213. doi: 10.1186/s40478-026-02449-x (PMC13660197; doi:10.1186/s40478-026-02449-x)
Supplement: Supplementary file 1 [file 40478_2026_2449_MOESM1_ESM.pdf]

Supplementary

Figure S1. Vascular cell collection and differentially expressed genes overlap.

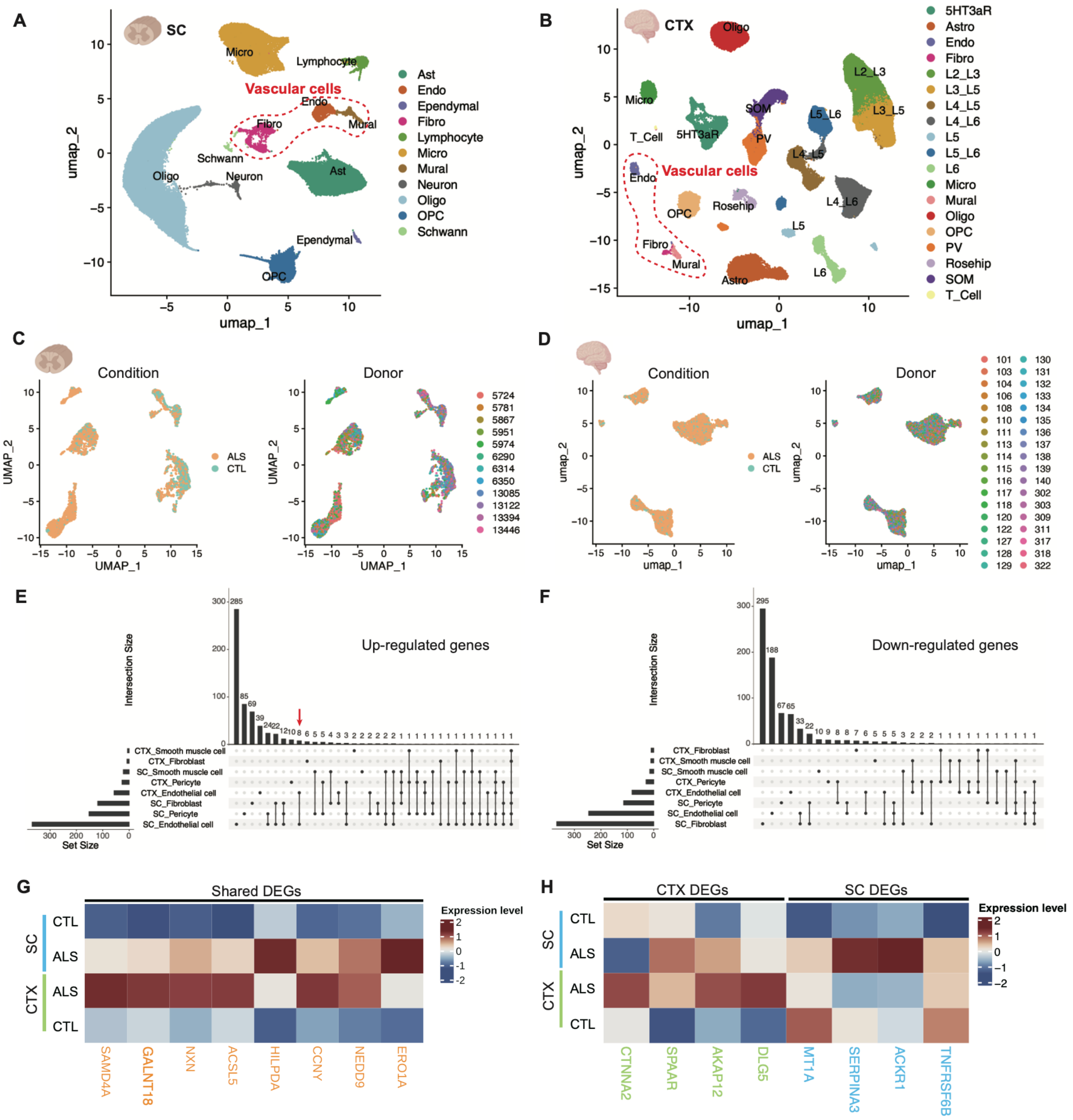

A, B. UMAP plots of all cell types in the spinal cord (**A**) and cortex (**B**). Vascular cells are highlighted. SC, spinal cord. CTX, cortex. **C, D**. UMAP plots showing the composition of different conditions and donors for the vascular atlas from the spinal cord (**C**) and cortex (**D**). **E, F**. Upset plots showing the up-regulated (**E**) and down-regulated (**F**) DEG overlap between two regions across 4 major classes. The arrow denotes the shared DEG amount of the endothelial class between the spinal cord and cortex. **G, H**. Heat maps showcasing shared (**G**) and region-specific (**H**) DEGs in endothelial cells between SC and CTX.

Figure S2. BSCB leakage in the *SOD1^{G93A}* mouse model.

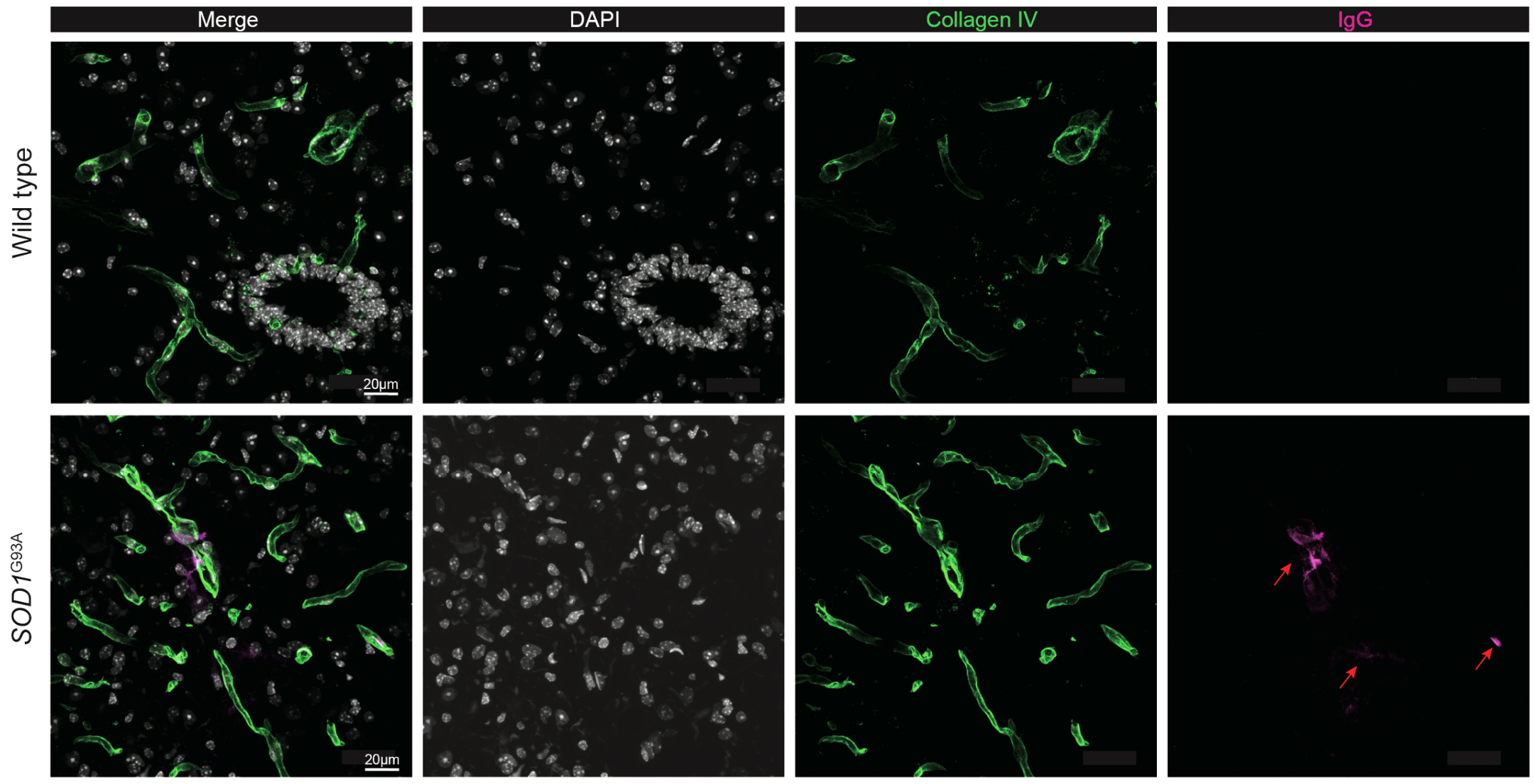

Immunofluorescence staining for Collagen IV and IgG in the spinal cord of 20-week-old wild type and *SOD1*^{G93A} transgenic mice.

Figure S3. Fibroblast heterogeneity in the spinal cord.

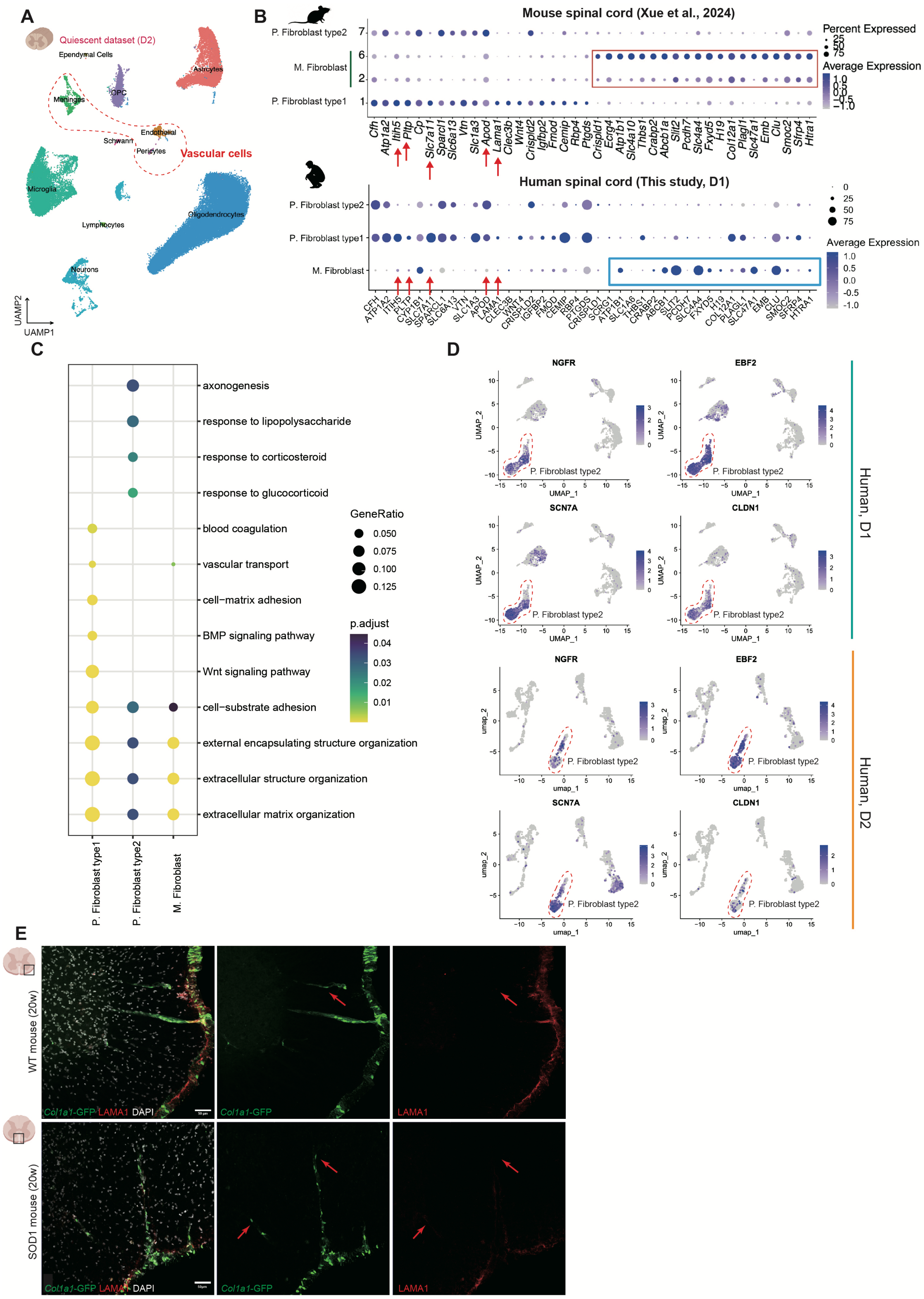

A. Healthy human spinal cord atlas from Yadav et al. Only the non-neuron cell population was presented here. **B**. Fibroblast gene expression in mouse (Up) and human (Down) spinal cord. Key P. Fibroblast markers are highlighted by red arrows. M. Fibroblast markers are highlighted in boxes. **C**. Gene ontology enrichment for DEGs from three fibroblast populations (D1). **D**. Feature plot showing the expression of major NAF markers in two

human spinal cord datasets. **E**. IF of *LAMA1* on different mouse replicates for both *SOD1*^{G93A} and wild type. Scale bar: 50μm.

Figure S4. Fibroblast gene expression and subtle activation in the cortex.

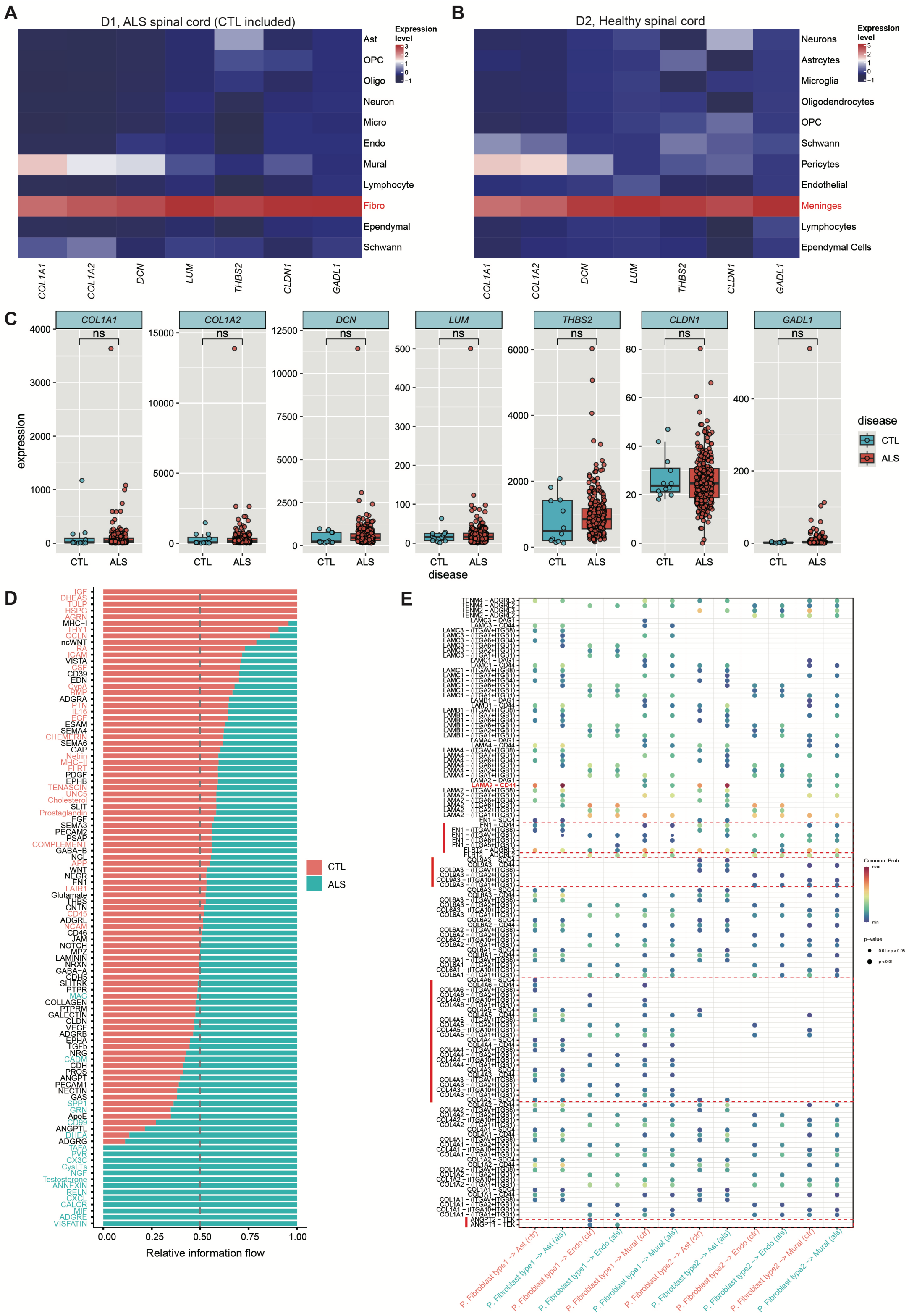

A, B. The expression of pan-fibroblast and type-specific markers within all cell types from both the ALS dataset (D1, left) and the quiescent dataset (D2, right). **C**. The expression of pan-fibroblast and type-specific markers in the cortex bulk RNA-seq dataset. **D**. The variation of different cell crosstalk pathways between ALS and healthy controls for all major cell types together. **E.** Communication probability of ECM-associated receptor-ligand pairs

from P. Fibroblast types to astrocytes (Ast), endothelial cells (Endo), and mural cells (Mural).
